# Self-rated health, life-style, and psychoendocrine measures of stress in healthy adult women

**DOI:** 10.3109/03009734.2010.496910

**Published:** 2010-10-27

**Authors:** Christina Halford, Lisa Ekselius, Ingrid Anderzen, Bengt Arnetz, Kurt Svärdsudd

**Affiliations:** ^1^Department of Public Health and Caring Sciences, Social Medicine Section, Uppsala University, UppsalaSweden; ^2^Department of Neuroscience, Psychiatry Section, Uppsala University, UppsalaSweden; ^3^Department of Public Health and Caring Sciences, Family Medicine and Clinical Epidemiology Section, Uppsala University, UppsalaSweden

**Keywords:** Cortisol, prolactin, self-rated health, sense of coherence, testosterone, vital exhaustion

## Abstract

**Background:**

Self-rated health (SRH) is a robust predictor of subsequent health outcome, independent of objective health measures and life-style-related health risk factors. However, the determinants of SRH are as yet largely unknown. In accordance with the prevailing stress theory, we hypothesized that SRH is associated with personal coping resources, psychological strain, life-style variables, and endocrine variables.

**Methods:**

A total of 106 healthy women, 22–59 years of age, were followed for up to 3 years with annual blood sampling (cortisol, prolactin, testosterone) and written questionnaires in which information on SRH, psychological strain, coping resources, socio-economic and life-style variables was sought.

**Results:**

In bivariate, screening logistic regression analyses, intended to find candidate variables for a final analysis model, all coping resource variables (sense of coherence, mastery, and self-esteem) were significantly related to SRH, and so were two psychological strain variables (vital exhaustion, and sleep disturbances), one life-style variable (fitness), but none of the endocrine variables. In the final multivariate analysis model, including all candidate variables, only vital exhaustion (*P* < 0.0001), fitness (*P* = 0.0002), and sense of coherence (*P* = 0.0006) were independently associated with SRH, together explaining 74% of the SRH variance.

**Conclusion:**

Some elements of the hypothesis, i.e. the effects of coping resources, psychological strain, and life-style variables on SRH, were supported by the results, while others, i.e. effects of endocrine measures on SRH, were not, indicating a possible gender difference.

Global self-ratings of health (SRH) are robust predictors of subsequent health outcomes, such as functional ability (1,2), health care utilization (3), morbidity (4,5), and mortality (6–9). Given the simplicity of use and predictive validity held, SRH has increasingly become used as an outcome measure in public health-based population surveys and health service evaluations*.*

A large body of research has been concerned with the question of what these simple SRH measures capture of importance to future health. Although attenuated, the association between SRH and health remains, even when objective health measures and known health risk factors are controlled for (6).

It has been suggested that stress theory-based psychobiological mechanisms may explain part of the predictive validity of SRH (6,10). SRH has been inversely associated with stress and positively associated with personal coping resource variables (11–14), and increasing evidence during the past decades suggests that sustained activation of the stress response systems may be associated with increased risk of disease (15–19).

The hypothalamic-pituitary-adrenal (HPA) axis is one of the primary effectors of the stress response systems. Chronic stress has been associated with increased levels of serum prolactin, decreased levels of serum testosterone, and with increased or decreased levels of serum cortisol, the latter having been suggested to occur following long-term exposure to chronic stressors (20–22).

Few studies investigating the suggested association between SRH and stress theory-based mechanisms have included concurrent measures of activity in the endocrine stress response, psychological strain, and personal coping resources (10,14,23,24). Moreover, as to our knowledge, there is only one previous study in which associations between endocrine measures of stress and SRH have been investigated specifically in women (25).

The aim of the present study was therefore to investigate associations between stress theory-based endocrine markers, psychological strain, personal coping resource variables, and SRH in a sample of healthy adult women. We hypothesized that SRH would be positively and independently associated with personal coping resources in terms of sense of coherence, mastery, and self-esteem, and with levels of testosterone, and negatively associated with psychological strain in terms of psychological symptoms, sleep disturbances, and vital exhaustion, and with levels of cortisol and prolactin.

## Methods

### Study population

The present study is part of a longitudinal project focusing on globalization of work, with a study population recruited as subjects or partners of subjects with planned assignments abroad, or as matched non-moving controls (26). The study population for this report consisted of the 107 women in the project. Results regarding associations between self-rated health and psychobiological markers of stress in men have been reported previously (24).

**Figure 1.**
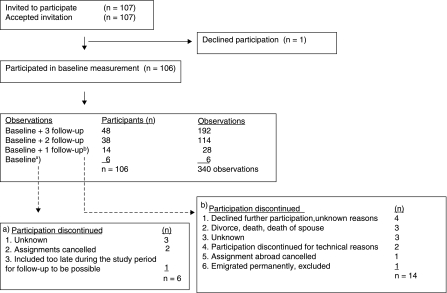
Flow chart of the study population.

Participants responded to a written questionnaire and had blood samples drawn at base-line and at 1-year intervals during the follow-up period, for a maximum of 3 years. The number of follow-up measurements performed varied, depending on the length of assignment abroad for the participants in the moving group, with the non-moving group followed for a corresponding period of time. The participation rate throughout the study was 81.1%. Reasons for non-participation at base-line and drop-out during follow-up are given in Figure 1.

### Questionnaire data

The written questionnaire included questions concerning SRH, personal coping resources, psychological strain, life-style factors, age, educational level, employment status, and medication. SRH was measured using a one-item global question phrased ‘How would say your general health has been during the past year?’ Possible responses ranged from ‘bad’ (=1) to ‘excellent’ (=5). Personal coping resources were assessed using the Pearlin 7-item mastery scale, which measures generalized beliefs about control (27), the Rosenberg 10-item scale assessing self-esteem (28), and Antonovsky's 13-item scale for assessment of sense of coherence (SOC) (29).

Psychological strain was measured using a 12-item version of the general health questionnaire (GHQ) scale (30). The 21-item version of Appel's Maastricht Questionnaire (31) was used to assess levels of vital exhaustion (VE), a construct conceptually similar to burn-out, which reflects a state of exhaustion thought to occur in response to prolonged stressor exposure (32). Assessment of sleep was based on a 6-item instrument consisting of a question concerning general sleep quality, with possible responses ranging from ‘very good’ (=1), to ‘very poor’ (=5), and further, questions concerning difficulties falling asleep, repeated night-time awakenings, nightmares, premature awakenings, and restless sleep, during the preceding six months, with possible responses ranging from ‘never’ (=1), to ‘every night’ (=5) (33). All instruments have previously been validity-tested (26).

**Table I.** Socio-economic characteristics of the study population.

	*n*	% or mean (SD)
Age, years		36.9 (9.3)
Self-rated health (*n* = 105), %		
Excellent	44	41.9
Good	43	41.0
Fair	12	11.4
Poor	3	2.9
Bad	3	2.9
Education (*n* = 104), %		
University	56	53.9
Secondary school	38	36.5
Compulsory school or vocational school	6	1.9
Other	4	7.6
Work (*n* = 106), %		
Full time	42	39.6
Part time	36	34.0
No gainful work	28	26.4
Children, <5 yrs old (*n* = 106), %	71	66.9
Exercise (*n* = 104), %		
Regularly, more than once a week	21	20.2
Regularly, once a week	25	24.0
Irregularly	56	53.8
Never	2	1.9
Fitness (*n* = 103), %		
Very good	5	4.9
Good	14	13.6
Average	65	63.1
Poor	17	16.5
Very poor	2	1.9
Current smoker (*n* = 104), %	17	16.3
Alcohol to relax after work (*n* = 104), %		
Daily	0	0
Once a week, but not every day	1	1.0
More than once a month, but less than once a week	27	26.0
Once a month or less	76	73.1
Medication (*n* = 101), %		
Daily^a^	2	2.0
Less often than daily^b^	4	3.9
No medication	95	94.1

^a^Anti-hypertensive medication.

^b^Analgesic medication (pain killers).

**Table II.** Psychoendocrine characteristics of the study population.

	Reference range or scale range	Mean	Median	Interquartile range
Endocrine measures				
Testosterone (nmol/L)	0.3–3.0	1.02	0.82	0.59–1.20
Cortisol (nmol/L)	230–700	379	358	282–450
Prolactin (μg/L)	3–19	13.6	6.8	4.6–11.0
Oestrogen (pmol/L)	0–1470^a^	1616.9	225.0	87.5–381.8
Coping resources, score				
Mastery	7–28	22.9	23.0	21.0–25.0
Self-esteem	10–40	33.0	34.0	31.0–36.0
Sense of coherence	13–91	70.1	72.0	64.0–77.0
Psychological strain, score				
GHQ	0–36	8.4	8.0	5.0–11.0
Sleep disturbances	6–30	12.0	11.0	9.0–15.0
Vital exhaustion	0–42	8.9	6.0	3.0–12.0

^a^Reference range for oestrogen: post-menopausal women 0–90 pmol/L; and for menstruating women, between 110–1470 pmol/L depending on menstrual cycle phase.

Leisure time exercise was classified on a scale ranging from ‘never’ (=1), to ‘regularly, more than once a week’ (=4). Self-rated fitness was classified on a scale ranging from ‘very poor’ (=1), to ‘very good’ (=5). Smoking habits were classified as currently being a smoker (=1) or a non-smoker (=0). Frequency of any type of alcohol intake in order to relax after work was classified on a scale ranging from ‘less than once a month’ (=1), to ‘daily’ (=4). For this report, educational level was classified as ‘compulsory or vocational school only’ (=1), ‘college’ (=2), ‘university level’ (=3), or ‘other’ (=4); employment status was classified as working ‘full time’ (=3), ‘part time’ (=2), or ‘no gainful work’ (=1); and medication was classified as ‘no medication’ (=0), ‘less than daily but at least once a week’ (=1), or ‘daily’ (=2).

### Blood samples

Venous blood samples were drawn between 8 a.m. and 10 a.m., following an overnight fast. They were centrifuged, and frozen at −20°C for later analysis at the Department of Clinical Chemistry, Karolinska University Hospital, which has an approved quality control/quality assurance (QC/QA) programme. Serum levels of prolactin and cortisol were analysed using time-resolved fluorescence immunoassay kits (AutoDELFIA, Wallac OY, Åbo, Finland). Serum testosterone levels were analysed using a RIA kit from Diagnostic Products Co (Los Angeles, USA). The coefficients of variation were 6.2% for prolactin, 8.2% for cortisol, and 12.8% for testosterone.

### Miscellaneous

An ordinal time variable was created to identify when blood and questionnaire data were collected (measurement occasion 1, 2, 3, and 4). Participants were categorized as pregnant, breast-feeding, or non-pregnant/non-breast-feeding, on each measurement occasion, based on verbal information in connection with blood sampling, on questionnaire data concerning number of children presently living in the household, and on levels of oestrogen and prolactin in blood sampled. The study was approved by the Research Ethics Committee at the Karolinska Institute, Stockholm (KI D No 91:28).

### Statistical considerations

Statistical analyses were performed using SPSS 13.0 (34) and SAS 9.1 (35) software. Data loss due to partial non-response (missing data in returned questionnaires or missing data in laboratory variables among participants) was 1.2% for questionnaire data, and 10.3% (range 9.4%–11.2%) for endocrine variables, mainly attributable to technical reasons. There were no significant differences in SRH between participants for whom endocrine data were or were not available. Psychological strain variables and the endocrine variables testosterone, prolactin, and oestrogen were highly skewed towards high values and were therefore log transformed before analysis.

In order to make full use of SRH as a five-level ordinal variable, multivariate ordinal logistic regression was used, providing pooled odds ratios (OR) across the five SRH levels for each independent variable, confidence intervals, and Wald's chi-square estimate (36). The last-mentioned one is the test parameter on which the *P*-value is based. As a consequence, Wald's chi-square estimate may be used to rank the impact, or importance, of the independent variables, and also to assess the contribution of the various levels to the total variance in multilevel analyses.

Change in SRH over time was non-isotonic, i.e. did not increase or decrease consistently across time, and the number of measurement occasions varied between subjects. To make full use of available data a cross-sectional analytical approach was used, based on a concatenated data arrangement, which means that data from each measurement occasion for a subject constituted an observation, or ‘data line’. Altogether 340 observations were produced by the 106 participating women, with their, on average, 3.2 measurement occasions.

A potential problem with this analytical approach is data dependence, since concatenated data are treated as if all measurements are independent, although up to four data lines may refer to the same subject. On the other hand, the measurement occasions were one year apart, diminishing the degree of dependence. Four methods were employed to check the degree of data dependence. First, an ordinal logistic regression-based multilevel analysis was performed on concatenated data, showing a very low degree of dependence. Furthermore, a cross-sectional analysis of base-line data only, an analysis based on mean values across time for each variable, and an analysis based on individual regression coefficients across time all showed similar results as those based on concatenated data, the only difference being that the concatenated data-based analysis had by far the highest statistical power. The last-mentioned was therefore used in this study.

Since a large number of potential independent variables, or SRH determinants, were involved, these were grouped into endocrine variables (testosterone, cortisol, and prolactin), life-style variables (fitness, exercise, smoking, and alcohol), personal coping resource variables (mastery, self-esteem, and sense of coherence), and psychological strain variables (GHQ, sleep, and vital exhaustion).

The analyses were performed in two steps. In the first, screening step candidate variables for further analysis were identified in each independent variable group, through multivariate ordinal logistic regression analyses, with SRH entered as outcome (dependent) variable, and the variables in each group entered as independent variables, one group at a time. Age, smoking and alcohol habits, pregnancy or breast-feeding, and oestrogen levels were entered as covariates, to adjust for their potential influence on outcome. In the second step, the significant candidate variables identified in the screening analyses were, together with the covariates, entered as independent variables in a final multivariate ordinal regression analysis model with backwards elimination of non-significant variables.

*Post hoc* power analyses based on the association of vital exhaustion and sense of coherence, on the one hand, and SRH on the other in this study, showed a beta of >90%, given the size of the study population, and an alpha of 0.05. For the effects of cortisol on SRH the statistical power was just short of 80%, while the power analyses of testosterone and prolactin on SRH indicated low power due to small effect size.

All tests were two-tailed. Significance levels were set at *P* < 0.05 in the screening analyses and at *P* < 0.01 in the final analyses model, to account for multiple testing. Confidence intervals were computed accordingly.

## Results

### Characteristics of the study population

Study-population characteristics are presented in Table I. Mean age was 37 years, range 22–59. At base-line, more than 80% rated their health as excellent or good. More than half had university education. A majority of the participants were married or co-habitants, in full-time or part-time work, and had children aged less than 5 years living in their household. Almost half of the participants reported regular exercise at least once a week, and a majority described their fitness as average. Less than one-fifth of the participants were smokers. The vast majority reported alcohol intake for relaxation purpose less than once a month. A total of 6% were on regular medication.

Data concerning endocrine measures, personal coping resources, and psychological strain variables are presented in Table II. The mean values were all well within the reference and scale ranges. All endocrine and psychological strain variables tended to be skewed towards high levels (mean higher than median), whereas the coping variables were fairly normally distributed.

### Screening analyses

Results of the screening analyses are shown in Table III. None of the endocrine variables showed any significant relationship with SRH and did thus not fulfil the candidate variable requirements. Among personal coping resource variables, all variables (sense of coherence, mastery, and self-esteem) fulfilled the candidate variable requirements, and so did vital exhaustion and sleep disturbances among psychological strain variables, whereas GHQ did not. Among the life-style variables, fitness but not exercise was significantly related to SRH and was thus chosen as candidate variable for the final regression analysis.

**Table III.** Multivariate screening analyses by variable group.

	OR	95% CI	Wald's chi-square	*P*
Endocrine variables^a^				
Testosterone^c^	1.51	0.51–4.50	0.6	0.46
Cortisol	1.00	1.00–1.00	0.1	0.78
Prolactin^c^	0.90	0.40–2.03	0.1	0.80
Coping resources^a^				
Sense of coherence	1.08	1.05–1.11	25.1	<0.0001
Mastery	1.17	1.06–1.30	8.8	0.003
Self-esteem	0.93	0.87–1.00	4.3	0.04
Psychological strain^a^				
Vital exhaustion^c^	0.23	0.11–0.51	13.1	0.0003
Sleep disturbances^c^	0.39	0.19–0.80	6.6	0.01
GHQ^c^	0.34	0.11–1.05	3.5	0.06
Life-style factors^b^				
Fitness	1.91	1.38–2.65	15.1	0.0001
Exercise	1.12	0.86–1.46	0.7	0.40

^a^Covariates = age, pregnancy, smoke, alcohol, oestrogen.

^b^Covariates = age, pregnancy, oestrogen.

^c^Based on log transformed values.

### Final multivariate ordinal regression analysis model

Based on the results of the screening analyses mastery, self-esteem, sense of coherence, vital exhaustion, sleep disturbances, and fitness were entered as independent variables into the final ordinal logistic regression analysis model with SRH as dependent variable (outcome) and age, smoking, alcohol in the evening in order to relax, on-going pregnancy/breast-feeding, and oestrogen entered as covariates. The results are presented in Table IV. Vital exhaustion, fitness and sense of coherence were, in descending order of importance according to Wald's chi-square estimate, independently associated with SRH at the *P* < 0.01 level. SRH was inversely associated with vital exhaustion and positively associated with fitness and sense of coherence. No other variable remained significantly associated with SRH. The final model explained 73.7% of the SRH variance.

**Table IV.** Final ordinal regression analyses model of the effects on self-rated health.^a^

	OR	99% CI	Wald's chi-square	*P*
Vital exhaustion^b^	0.24	0.12–0.48	16.3	<0.0001
Fitness	1.73	1.30–2.31	13.8	0.0002
Sense of coherence	1.05	1.02–1.08	11.6	0.0006

^a^The following non-significant variables were eliminated by the backward elimination procedure: on-going pregnancy/breast-feeding (*P* = 0.90), oestrogen (*P* = 0.59), alcohol (*P* = 0.39), age (*P* = 0.23), sleep disturbances (*P* = 0.13), self-esteem (*P* = 0.10), smoking (*P* = 0.5), and mastery (*P* = 0.02).

^b^Based on log transformed values.

### Visualization of the final analysis model

**Figure 2.**
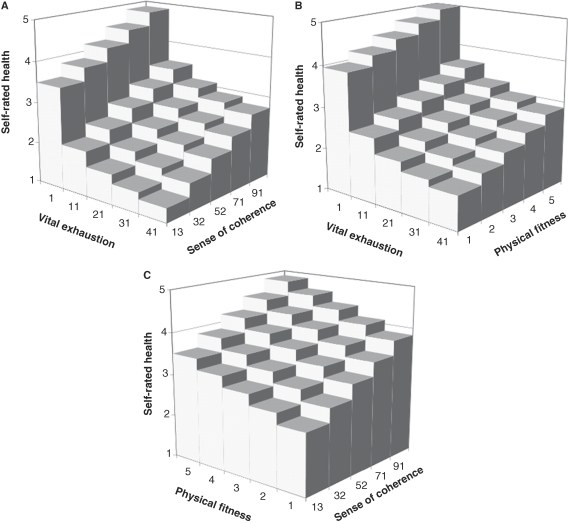
Effects on self-rated health of various combinations of vital exhaustion, fitness, and sense of coherence.

The independent effects of vital exhaustion, fitness, and sense of coherence on SRH are visualized in Figure 2. The lowest level of SRH (=1) was found for the highest level of vital exhaustion (=41) combined with the lowest level of sense of coherence (=13) (Figure 2A). The SRH range was 1.3–4.9 for various combinations of vital exhaustion and sense of coherence. The combined effects on SRH of vital exhaustion and physical fitness are shown in Figure 2B, and of physical fitness and sense of coherence in Figure 2C.

## Discussion

There was a strong association between vital exhaustion, fitness, and sense of coherence on the one hand, and SRH on the other, with the final regression model explaining almost 74% of the observed SRH variance. No statistically significant associations were observed between endocrine variables and SRH. To our knowledge, this is the first study investigating associations between endocrine as well as psychological stress theory-based variables and SRH, in healthy middle-aged women.

The study takes its departure point in the hypothesis that SRH may be viewed as a potential risk indicator of sustained responses to stressor exposure, and that stress theory-based mechanisms explain part of the predictive validity of SRH in relation to future health. An important question concerns the validity of independent variables investigated in relation to the research question posed. Face validity of vital exhaustion and sense of coherence may, from a stress theory-based perspective, be considered good. Vital exhaustion, a state of exhaustion thought to occur in response to prolonged psychological stress, is conceptually similar to burn-out, and empirically close correlations with various burn-out measures have been reported (16). The SOC-questionnaire was distinctly developed as a stress theory-based construct (29).

Regarding criterion validity, vital exhaustion has, in longitudinal studies, independent of known risk factors for cardiovascular disease and severity of concurrent disease, been associated with increased risk of cardiovascular disease and with all-cause mortality (16,37,38). Sense of coherence has in longitudinal studies been found inversely associated with risk of diabetes (39), levels of all-cause mortality (40,41), and with coronary heart disease in white-collar workers but not among blue-collar workers (42). Longitudinal studies are, however, as yet few.

Mean and median levels of vital exhaustion in the present study corresponded to levels previously reported in healthy study populations (38). Mean level of sense of coherence was slightly higher than levels previously reported in Swedish women with similar socio-demographic characteristics (43).

There was a strong inverse association between psychological strain, in terms of vital exhaustion, and SRH. Results are in line with observations concerning healthy adult men based on data from the same original study population (24) and in line with recently reported observations from a cross-sectional study exploring the relationship between vital exhaustion, personal coping resources, and SRH, in adult men (14). There are, to our knowledge, no previous studies investigating associations between VE and SRH in healthy women.

In line with previous research there was a strong positive effect of personal coping resources, in terms of sense of coherence, on SRH, (14,44). Results furthermore suggest that mastery (locus of control) and self-esteem represent lower-order personal coping resources with weaker effect on SRH than does SOC.

Importantly there were no statistically significant associations between endocrine variables and SRH. *Post hoc* power analyses indicated a low statistical power in the analyses of testosterone and prolactin on the one hand, and SRH on the other, not due to a too-small study population but to a too-small effect size, indicating that the variables in question may be associated, but that the direct effect of the independent variable on the dependent variable is weak. Applied to the present study, results thus suggest that testosterone and prolactin may be associated with SRH, but that the associations perhaps are mediated by other variables, attenuating the direct relationship. Associations between prolactin, testosterone, and SRH have previously been reported in healthy adult men, based on data from the same original study (24), one of few in this field*.* Bivariate associations between prolactin and SRH were observed in a recent study of adult women (25). The presence of associations between SRH and endocrine variables in men but not in women may reflect gender differences.

There were no significant associations observed between cortisol and SRH, which is in line with results from two previous studies based on male study populations (14,24). However, the statistical power observed for the cortisol–SRH relation was in the present study just short of 80%, suggesting that lack of effect may be attributable to a too-small study population. Results concerning cortisol should thus be interpreted with caution.

Associations between life-style factors and SRH are well established. In the present study, perceived fitness was a strong determinant of SRH. Results thus underline the importance of including life-style variables in regression models aimed at understanding the role of potential predictors of SRH. Contrary to expectations, smoking was positively associated with SRH.

Limitations of the study concern questionnaire data on some of the life-style-related variables. For smoking and alcohol consumption, data were somewhat crudely measured. Similarly, endocrine data were based on sampling performed once on each measurement occasion; for increased precision, repeated blood sampling at each measurement point in time would have been desirable. Furthermore, associations between SRH and salivary, but not serum, cortisol levels have been reported (14), raising important questions concerning effects of different sampling methodology on results concerning cortisol.

The main strengths of the study include that the study population consisted of healthy women with low levels of health risk behaviours. Associations observed between independent variables and SRH can thereby not be explained in terms of effects of concurrent disease or disability*.* A further strength of the study concerns the use of ordinal regression modelling (36,45). A majority of previous studies investigating predictors of five-level SRH have either been based on binary logistic regression technique, despite the reduced efficiency and the risk of bias related to choice of cut point this entails, or on analytic methods which require normally distributed SRH data, an assumption rarely met. The analytical methodology used in this study provided excellent power in spite of a fairly moderate study population size, owing to the high precision obtained by the concatenated data structure.

Analyses were cross-sectional, and causal inferences can therefore not be made. Repeated studies, based on larger study populations, and repeated blood sampling, with results reported separately for women and men, are required, to test the reliability of the present results. Finally, the present study was focused on investigating associations between stress theory-based psychobiological variables and SRH. Future studies need to determine if, and to what extent, observed associations contribute to the predictive validity of SRH in relation to future health.

## Conclusions

Results supported the hypothesis that personal coping resources and psychological strain variables affect SRH but gave no evidence of associations between endocrine variables and SRH in this sample of healthy adult women.
